# Single trial somatosensory evoked potential extraction with ARX filtering for a combined spinal cord intraoperative neuromonitoring technique

**DOI:** 10.1186/1475-925X-6-2

**Published:** 2007-01-04

**Authors:** Lorenzo Rossi, Anna Maria Bianchi, Anna Merzagora, Alberto Gaggiani, Sergio Cerutti, Francesco Bracchi

**Affiliations:** 1 Department of Human Physiology, University of Milan, Italy; 2 Department of Biomedical Engineering, Polytechnic of Milan, Italy; 3 School of Biomedical Engineering, Science and Health Systems, Drexel University, Philadelphia, PA, USA

## Abstract

**Background:**

When spinal cord functional integrity is at risk during surgery, intraoperative neuromonitoring is recommended. Tibial Single Trial Somatosensory Evoked Potentials (SEPs) and H-reflex are here used in a combined neuromonitoring method: both signals monitor the spinal cord status, though involving different nervous pathways. However, SEPs express a trial-to-trial variability that is difficult to track because of the intrinsic low signal-to-noise ratio. For this reason single trial techniques are needed to extract SEPs from the background EEG.

**Methods:**

The analysis is performed off line on data recorded in eight scoliosis surgery sessions during which the spinal cord was simultaneously monitored through classical SEPs and H-reflex responses elicited by the same tibial nerve electrical stimulation. The single trial extraction of SEPs from the background EEG is here performed through AutoRegressive filter with eXogenous input (ARX). The electroencephalographic recording can be modeled as the sum of the background EEG, which can be described as an autoregressive process not related to the stimulus, and the evoked potential (EP), which can be viewed as a filtered version of a reference signal related to the stimulus. The choice of the filter optimal orders is based on the Akaike Information Criterion (AIC). The reference signal used as exogenous input in the ARX model is a weighted average of the previous SEPs trials with exponential forgetting behavior.

**Results:**

The moving average exponentially weighted, used as reference signal for the ARX model, shows a better sensibility than the standard moving average in tracking SEPs fast inter-trial changes. The ability to promptly detect changes allows highlighting relations between waveform changes and surgical maneuvers. It also allows a comparative study with H-reflex trends: in particular, the two signals show different fall and recovery dynamics following stressful conditions for the spinal cord.

**Conclusion:**

The ARX filter showed good performances in single trial SEP extraction, enhancing the available information concerning the current spinal cord status. Moreover, the comparison between SEPs and H-reflex showed that the two signals are affected by the same surgical maneuvers, even if they monitor the spinal cord through anatomically different pathways.

## Background

The monitoring of the functionality of vital parameters is known to be a basic aspect in up-to-date surgical techniques. This is particularly true in the case of surgery performed on the vertebral column (i.e.: correction of serious scoliosis), when a continuous monitoring of the spinal cord functionality is usually required [1-4].

In fact, during surgery the integrity and functionality of the spinal cord may be compromised by surgical maneuvers to such an extent as to cause prolonged insufficient blood supply to the cord, or mechanical compression and, as a consequence, its temporary malfunctioning. Sometimes this malfunctioning, if undetected at an early stage, can lead to irreversible spinal cord damage [5-7].

**Figure 1:**
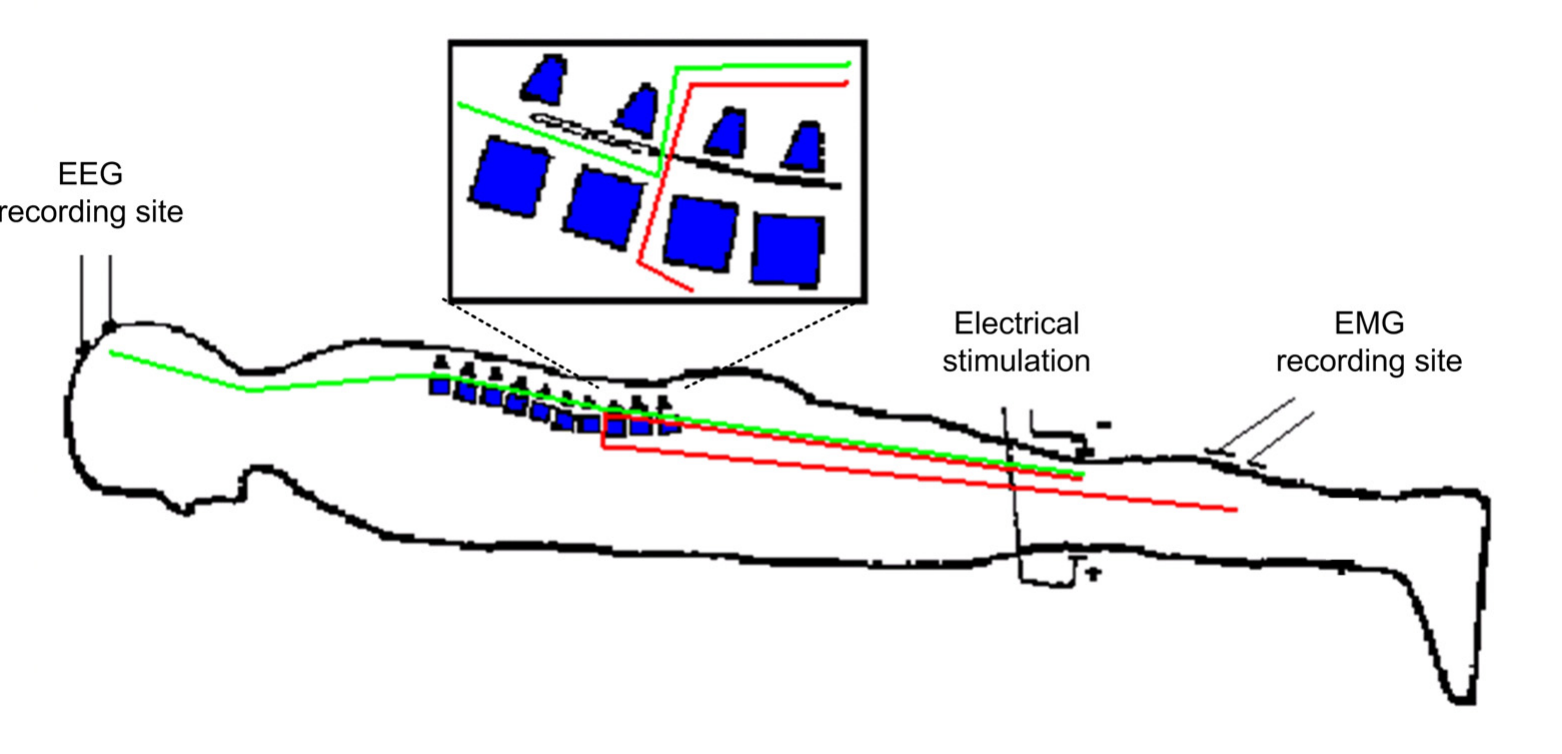
**Applied neuromonitoring technique**. The stimulation involves the somatosensory pathway, plotted in green, and the H-reflex pathway, plotted in red.

The aim of this study is to present a signal processing method used for extracting a single trial potential in a new combined neuromonitoring technique [8], based on the simultaneous monitoring of parameters extracted from two neurophysiologic signals: the single trial somatosensory evoked potentials (SEPs) [9,10] and the soleus muscle H-reflex [11,12], both elicited by the same electrical stimulus to the posterior tibial nerve (Fig. 1). The advantage of observing two different signals both elicited by the same stimulus, instead of observing the variation of only one of them, is that the variability of the monitoring is decreased. Moreover the SEP provides information on the functionality of the ascending pathways, up to the somatosensory cortex, while the H-reflex involves mainly the descending motor pathways. Thus the pieces of information coming from the two systems are complementary, and the use of both can provide a more reliable and robust monitoring, especially when real-time processing and immediate decisions are required as in a surgical theatre. Indeed the SEP and the H-reflex involve different nervous fibers which however share the same peripheral nerve and both depend on the integrity of spinal cord pathways. It is therefore reasonable to expect that these two signals should show an independent variability but a damage to the peripheral or medullar function would modify the two signals in the same direction hence, it is hypothesized that the real time comparison of the SEP and the H-reflex, both elicited by the same stimulus, should significantly increase the monitoring reliability and sensitivity, as suggested in [13-15].

However, whereas the H-reflex analysis does not require refined processing methods, the SEPs due to their poor signal to noise ratio (SNR), require proper tools for the analysis of the EEG in order to extract the evoked potential waveform.

The current clinical SEP extraction techniques usually average several trials in order to enhance the SNR. However, the SEP has a trial-to-trial variability which is lost if it is extracted using the averaging technique. This would lead to the loss of information about the trial-to-trial changes of the SEP signal.

When combining SEPs and H-reflex, a limit is introduced by the long recovery time of the H-reflex, so that the inter-stimulus interval (ISI) must be at least 10 s [15]-[16]. In case of a SEPs' sudden changes the averaging technique provides intolerably delayed information, for this reason, a single trial SEP analysis is needed in order to compare it with the H-reflex obtained from the same electrical stimulus.

## Methods

### Clinical protocol

In this study, recordings from 8 scoliosis correction surgery cases are utilized. The correction was performed at the same spinal level with similar surgical procedures: 6 of them were corrected with Cotrel-Dubousset technique; the other 2 were corrected with the Harrington technique (Table 1). In all cases the same i.v. Propofol anesthesia was used. All analyzed cases did not present any post operative complication after recovery from anesthesia.

**Table 1:** Surgery sessions monitored

Age	Sex	Level of malformation	Surgery correction technique
31	F	D5-L4	CD
13	F	D5-L1	HR
17	M	D3-L3	HR
20	F	D5-L3	CD
14	F	D4-L4	CD
17	F	D3-L1	CD
16	F	D3-L4	CD
53	F	D4-L5	CD

CD is for Cotrel-Dubousset, HR is for Harrington.

EEG trials for the evoked potentials and EMG for the H-reflex were acquired during surgery form each of eight patients. The signals were elicited stimulating the posterior tibial nerve with a 1 ms rectangular voltage pulse up to 150 V at 0.1 Hz repetition rate.

The EEG signal was recorded through needle electrodes placed on the scalp in Cz' with an auricular reference. The H-reflex was acquired using a surface electrode (RedDot, 3 M, USA) on the soleus muscle and a reference one over the Achilles tendon.

The EEG and EMG acquisition were performed by means of two analog ampli-filters ICP 511-AP by Grass Instruments, with different gains and filter settings (EEG: gain = 80dB, bandpass 1 Hz – 300 Hz; EMG: gain = 40 dB, bandpass 30 Hz – 3 kHz).

The stimulus triggered EMG recording lasts 62.5 ms, sampled at 10 kHz; the EEG recording lasts 125 ms, sampled at 2.5 kHz by PCI board (type PCI-MIO-16E-4, National Instruments, USA). The acquisition board range is +/-1.25 V with a 12-bit resolution.

The analysis software used for off-line processing the recorded data has been implemented by means of Virtual Instruments developed in LabView with integrated Matlab processing scripts. The software performs the single trial extraction of SEP waveform and the detection of amplitude and latency both of the SEP and of the H-Reflex simulating an on-line neuromonitoring system.

### EEG signal processing

#### ARX model

Many algorithms have been proposed in order to obtain a single trial SEP from the EEG [17-22] or for improving the signal to noise ratio for neuromonitoring application.

An Autoregressive Model with an eXogenous Input (ARX) has been chosen, because it has been successfully applied and tested in the analysis of different kinds of evoked potentials (visual, acoustic, somatosensory [23-25]).

According to the ARX model, the signal *y*(*k*), recorded after a somatosensory stimulation, can be modeled as the sum of two different contributions: a former not related to the stimulus (the background EEG) that, according to the literature [26], can be described as an autoregressive process driven by a white noise *e*(*k*); a latter related to the stimulus (the EP), that can be viewed as a filtered version of a reference signal *u*(*k*). The general equation of an ARX process is:

y(k)=−∑i=1nai⋅y(k−i)+∑j=dm+d−1bj⋅u(k−j)+e(k)     (1)
 MathType@MTEF@5@5@+=feaafiart1ev1aaatCvAUfKttLearuWrP9MDH5MBPbIqV92AaeXatLxBI9gBaebbnrfifHhDYfgasaacH8akY=wiFfYdH8Gipec8Eeeu0xXdbba9frFj0=OqFfea0dXdd9vqai=hGuQ8kuc9pgc9s8qqaq=dirpe0xb9q8qiLsFr0=vr0=vr0dc8meaabaqaciaacaGaaeqabaqabeGadaaakeaacqWG5bqEcqGGOaakcqWGRbWAcqGGPaqkcqGH9aqpcqGHsisldaaeWbqaaiabdggaHnaaBaaaleaacqWGPbqAaeqaaaqaaiabdMgaPjabg2da9iabigdaXaqaaiabd6gaUbqdcqGHris5aOGaeyyXICTaemyEaKNaeiikaGIaem4AaSMaeyOeI0IaemyAaKMaeiykaKIaey4kaSYaaabCaeaacqWGIbGycqWGQbGAcqGHflY1cqWG1bqDcqGGOaakcqWGRbWAcqGHsislcqWGQbGAcqGGPaqkaSqaaiabdQgaQjabg2da9iabdsgaKbqaaiabd2gaTjabgUcaRiabdsgaKjabgkHiTiabigdaXaqdcqGHris5aOGaey4kaSIaemyzauMaeiikaGIaem4AaSMaeiykaKIaaCzcaiaaxMaadaqadaqaaiabigdaXaGaayjkaiaawMcaaaaa@672C@

The model orders (n, m, d) are defined as follows:

• *n *and *m*, that are respectively the model orders of the autoregressive and of the moving average sections;

• *d*, takes into account the possible temporal anticipation between the reference input and the output of the filter (the filter is not causal).

In the z-transform domain, where z^-1 ^is the unit delay operator, the ARX model is expressed as:

*A *(*z*)·*Y *= *B *(*z*)·*U *+ *E *    (2)

and

Y=B(z)A(z)⋅U+EA(z)     (3)
 MathType@MTEF@5@5@+=feaafiart1ev1aaatCvAUfKttLearuWrP9MDH5MBPbIqV92AaeXatLxBI9gBaebbnrfifHhDYfgasaacH8akY=wiFfYdH8Gipec8Eeeu0xXdbba9frFj0=OqFfea0dXdd9vqai=hGuQ8kuc9pgc9s8qqaq=dirpe0xb9q8qiLsFr0=vr0=vr0dc8meaabaqaciaacaGaaeqabaqabeGadaaakeaacqWGzbqwcqGH9aqpdaWcaaqaaiabdkeacjabcIcaOiabdQha6jabcMcaPaqaaiabdgeabjabcIcaOiabdQha6jabcMcaPaaacqGHflY1cqWGvbqvcqGHRaWkdaWcaaqaaiabdweafbqaaiabdgeabjabcIcaOiabdQha6jabcMcaPaaacaWLjaGaaCzcamaabmaabaGaeG4mamdacaGLOaGaayzkaaaaaa@44F0@

**Figure 2:**
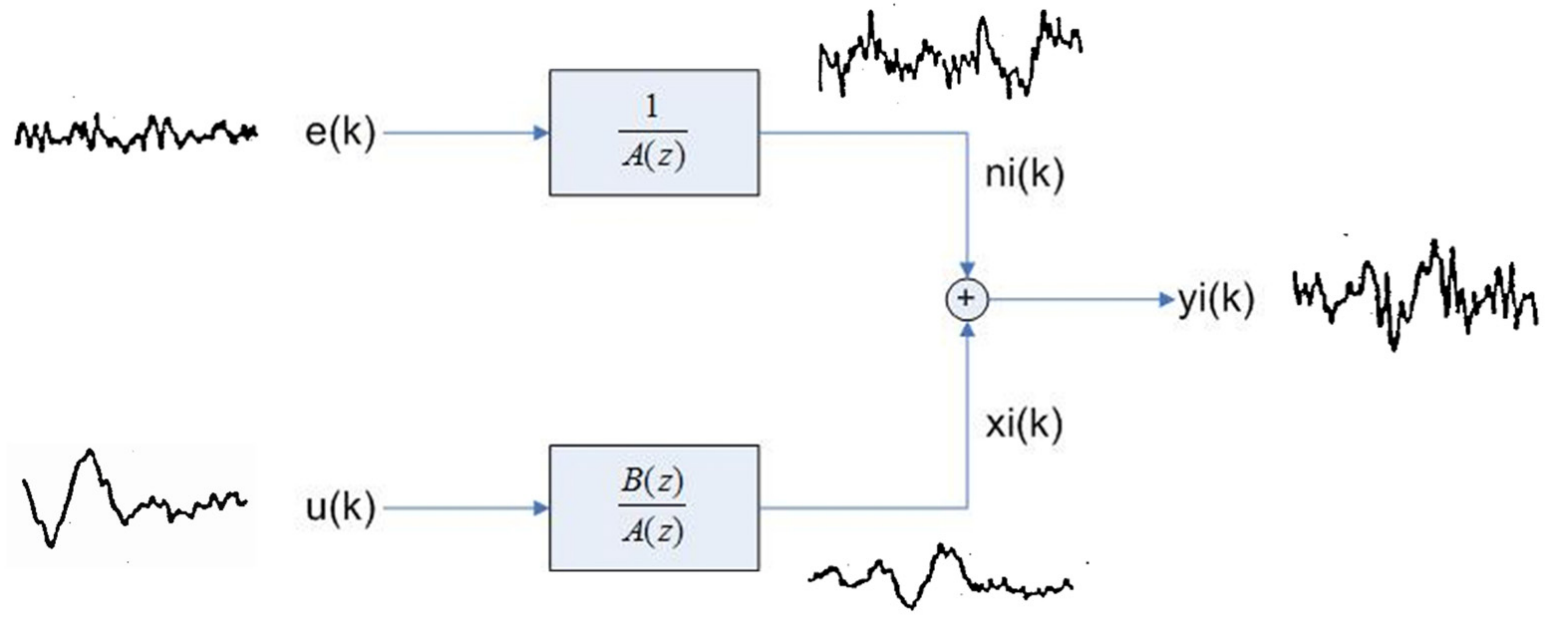
ARX model applied to the single trial evoked potential extraction.

This last expression (3) is visualized by the block diagram shown in Fig. 2.

If *u*(*k*) and *y*(*k*) are known, then the *a*_*i *_and *b*_*j *_coefficients can be estimated together with the variance of the input white noise, for every pre-fixed model order set and delay (*n*, *m*, *d*), by means of a least squares approach, that minimizes the following figure of merit:

J=1N∑1N[err(k)]2     (4)
 MathType@MTEF@5@5@+=feaafiart1ev1aaatCvAUfKttLearuWrP9MDH5MBPbIqV92AaeXatLxBI9gBaebbnrfifHhDYfgasaacH8akY=wiFfYdH8Gipec8Eeeu0xXdbba9frFj0=OqFfea0dXdd9vqai=hGuQ8kuc9pgc9s8qqaq=dirpe0xb9q8qiLsFr0=vr0=vr0dc8meaabaqaciaacaGaaeqabaqabeGadaaakeaacqWGkbGscqGH9aqpdaWcaaqaaiabigdaXaqaaiabd6eaobaadaaeWbqaamaadmaabaGaemyzauMaemOCaiNaemOCaiNaeiikaGIaem4AaSMaeiykaKcacaGLBbGaayzxaaWaaWbaaSqabeaacqaIYaGmaaaabaGaeGymaedabaGaemOta4eaniabggHiLdGccaWLjaGaaCzcamaabmaabaGaeGinaqdacaGLOaGaayzkaaaaaa@435C@

where err(k) is the prediction error of the model and N is the number of samples in the trial. The prediction error is the difference between the recorded signal and its prediction according the model expressed in eq. 1.

#### Choice of the model order set (*n*, *m*, *d*)

The method used for selecting the optimal model order set has been implemented in the following steps:

- Choice of an initial set of model orders (*n*, *m*, *d*);

- Minimization of a figure of merit *J *in order to find the best ARX coefficients for the chosen order.

- Check the whiteness of prediction error *err*(*k*): the set of identified parameters is accepted for the following calculations when the *err*(*k*) is a white sequence with a confidence level of 95%; the whiteness of the residual is tested using the cumulative Anderson test.

- If the whiteness test is not verified, the model orders are increased until the prediction error is a white noise.

The starting triplet (*n*, *m*, *d*), selected in the first step, is calculated for each subject before starting the monitoring phase of the risky surgical procedures.

The first 50 sweeps are recorded and stored, and the optimal model orders, among the ones that satisfy the whiteness criterion, are selected by minimizing the Akaike Information Criterion (AIC) function; though there are several methods for finding the optimal orders for model (Final Prediction Error, Minimum Description Length, etc.), for the specific application the best one is the AIC because where the model orders are low the AIC shows better performances [27].

The AIC function is expressed as:

aic=ln⁡(σ2)+n+mN     (5)
 MathType@MTEF@5@5@+=feaafiart1ev1aaatCvAUfKttLearuWrP9MDH5MBPbIqV92AaeXatLxBI9gBaebbnrfifHhDYfgasaacH8akY=wiFfYdH8Gipec8Eeeu0xXdbba9frFj0=OqFfea0dXdd9vqai=hGuQ8kuc9pgc9s8qqaq=dirpe0xb9q8qiLsFr0=vr0=vr0dc8meaabaqaciaacaGaaeqabaqabeGadaaakeaacqWGHbqycqWGPbqAcqWGJbWycqGH9aqpcyGGSbaBcqGGUbGBdaqadaqaaGGaciab=n8aZnaaCaaaleqabaGaeGOmaidaaaGccaGLOaGaayzkaaGaey4kaSYaaSaaaeaacqWGUbGBcqGHRaWkcqWGTbqBaeaacqWGobGtaaGaaCzcaiaaxMaadaqadaqaaiabiwda1aGaayjkaiaawMcaaaaa@426F@

where *σ*^2 ^is the variance of the prediction error *err*(*k*).

The *d *parameter is chosen as m/2 considering a previous investigation [18].

On the basis of the AIC function, evaluated on the first 50 recorded sweeps, the triplet (*n*, *m*, *d*) is selected for the specific patient.

#### The reference signal

In many applications of the ARX filter for the extraction of the single trial EP, the reference *u*(*k*) is the average of a sufficient number of trials, representing a standard EP shape. The choice of the reference signal is fundamental for the ARX filter capability in tracking the inter-trials EP variations: in fact the filter tries to identify a waveform, similar to the input reference, in the recorded signal trial y(t). Thus, a fixed reference could be misleading in the case of a dynamic process. For this reason a fast adapting reference could be a better choice in order to be able to track also sudden changes in the waveform.

However, the ability of this reference to adapt to morphological variations in the EP wave depends on the number of sweeps included: the moving average introduces a delay in the updating of the reference that depends on the number of averaged trials. In order to enhance the adaptation ability of the reference signal, a moving average with an exponential decay is here proposed and tested.

The reference is computed as follows:

*u*_*i *_= *μ*·*u*_*i *- 1 _+ (1 - *μ*)·*y*_*i *_    (6)

The *μ *coefficient (*forgetting coefficient*) provides an exponential windowing on the averaged sweeps, so that only the most recent trials contribute significantly to the reference *u*_*i*_, while the oldest ones are progressively forgotten, with an exponential decay. Different values of *μ *have been tested in order to have a reference signal that adaptively varies according to the possible dynamical changes in the evoked potentials. Off-line tests led to the value *μ *= 0.95 [28].

In order to compare the ARX performances with the reference built on a static average and with reference built on a moving average driven by a forgetting coefficient, as defined by the eq. (6), signal processing simulations have been performed.

Three known input signals, representing the *x*_*i*_(*k*) target signals, have been built from one generic EP that has been multiplied for 3 different coefficients: *k*_*1 *_= 20, *k*_*2 *_= 15, *k*_*3 *_= 1 in order to simulate different amplitudes.

An AR model has been identified from an EEG trial of 125 ms not related to any stimulus. A white Gaussian noise has been utilized as the AR model input in order to generate EEG signals *n*_*i*_(*k*). With this method it was possible to collect, as the sum of random *n*_*i*_(*k*) and known targets signals k_j_*x_*i*_(*k*), 50 trials using the *k*_*1*_coefficient, 1 using the *k*_*2 *_and 1 using the *k*_*3*_. These input signals have been utilized for simulating a possible variation of the EP due to a stressful maneuver applied at the 51^st ^trial that produces a strong EP decrease at the 52^nd ^trial.

The ARX filter using the two different reference signals, i.e. the moving average and the moving average with forgetting coefficient, have been compared according to their ability to extract the input signals k_j_*x_*i*_(*k*).

#### Correlation with the H-reflex amplitude

The degree of correlation between the amplitude of SEP and H-reflex has been computed through the Pearson's correlation coefficient in the 8 surgery sessions studied.

The Pearson's coefficient is defined as:

r=∑(As−As˜)⋅(Ah−Ah˜)n−1Ss⋅Sh     (7)
 MathType@MTEF@5@5@+=feaafiart1ev1aaatCvAUfKttLearuWrP9MDH5MBPbIqV92AaeXatLxBI9gBaebbnrfifHhDYfgasaacH8akY=wiFfYdH8Gipec8Eeeu0xXdbba9frFj0=OqFfea0dXdd9vqai=hGuQ8kuc9pgc9s8qqaq=dirpe0xb9q8qiLsFr0=vr0=vr0dc8meaabaqaciaacaGaaeqabaqabeGadaaakeaacqWGYbGCcqGH9aqpdaWcaaqaamaalaaabaWaaabqaeaacqGGOaakcqWGbbqqcqWGZbWCcqGHsislcqWGbbqqcuWGZbWCgaacaiabcMcaPiabgwSixlabcIcaOiabdgeabjabdIgaOjabgkHiTiabdgeabjqbdIgaOzaaiaGaeiykaKcaleqabeqdcqGHris5aaGcbaGaemOBa4MaeyOeI0IaeGymaedaaaqaaiabdofatjabdohaZjabgwSixlabdofatjabdIgaObaacaWLjaGaaCzcamaabmaabaGaeG4naCdacaGLOaGaayzkaaaaaa@513A@

where A_s _and A_h _are the amplitudes of SEP and H-reflex, *A*s˜
 MathType@MTEF@5@5@+=feaafiart1ev1aaatCvAUfKttLearuWrP9MDH5MBPbIqV92AaeXatLxBI9gBaebbnrfifHhDYfgasaacH8akY=wiFfYdH8Gipec8Eeeu0xXdbba9frFj0=OqFfea0dXdd9vqai=hGuQ8kuc9pgc9s8qqaq=dirpe0xb9q8qiLsFr0=vr0=vr0dc8meaabaqaciaacaGaaeqabaqabeGadaaakeaacuWGZbWCgaacaaaa@2E2A@ and *A*h˜
 MathType@MTEF@5@5@+=feaafiart1ev1aaatCvAUfKttLearuWrP9MDH5MBPbIqV92AaeXatLxBI9gBaebbnrfifHhDYfgasaacH8akY=wiFfYdH8Gipec8Eeeu0xXdbba9frFj0=OqFfea0dXdd9vqai=hGuQ8kuc9pgc9s8qqaq=dirpe0xb9q8qiLsFr0=vr0=vr0dc8meaabaqaciaacaGaaeqabaqabeGadaaakeaacuWGObaAgaacaaaa@2E14@ are the averages of the respective amplitudes, S_s _and S_h _are the standard deviations and *n *is the number of the considered trials. Pearson's coefficient value indicates the correlation level for a specific freedom degree given by the number of trials analyzed; a coefficient value of 1 indicates a perfect correlation whereas a value of 0 indicates no correlation.

### Data analysis protocol

The sequence of the off-line data analysis steps have been structured in order to comply with the requirements for a data processing during a surgery session.

The data analysis steps proposed are:

- *Cleaning corrupted recorded trials*

This procedure automatically deletes the trials corrupted by environmental noise by checking 2 different parameters:

1. presence of saturated samples: if there is at least one sample with a value of +/-1.25 V (the maximum/minimum acquisition board voltage input), the trial is rejected;

2. single-trial amplitude range: given the maximum sample value (Max) and the minimum sample value (Min) of the *ith*-trial, if |Max-Min|>0.7 V, the trial is rejected.

All the parameter checking and its respective threshold values have been experimentally found.

- *Building the initial reference signal*

The first reference signal is obtained from 50 trials recorded before the beginning of risky surgical maneuvers. Then, the reference signal is updated at each trial throughout the surgery according to the weighted moving average procedure described above.

- *Choosing the optimal order for the model*

The trial set, used to build the initial reference signal, is also used to evaluate the best model order. The following range of model orders have been used in order to calculate the AIC function:

[1<n<21], [2<m<20] and d = m/2.

- *Single sweep analysis*.

The trials acquired during the surgical procedure are evaluated in combination with the H-reflex signal.

## Results

### Simulations

**Figure 3:**
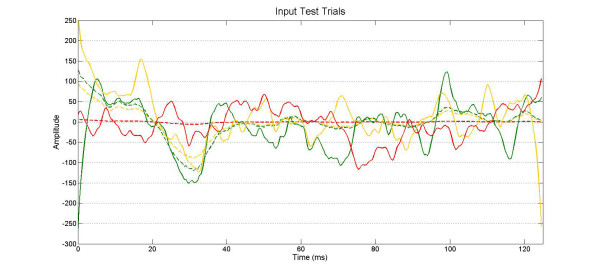
**Input test signals**. The waveforms plotted in dashed lines are the test inputs k_j_*x_*i*_(*k*) (*k*_1 _= 20, *k*_2 _= 15, *k*_3 _= 1; see text for details) summed to the random n_i_(k) EEG noise in order to generate the simulated raw trials y_i_(k), plotted in bold lines. The green dashed and bold lines are related to the 50^th ^simulated y_50_(k) raw trial, the yellow ones to the 51^ft ^trial and the red ones to the 52^nd ^trial.

The 3 input test trials computed by the AR model built from a generic EEG trial have been utilized as target input signals for single trials extracting simulation (Fig. 3). It is very difficult to valuate without any signal processing that the 52^nd ^unfiltered trial has a known input EP signal one tenth smaller than the 50^th ^trial.

**Figure 4:**
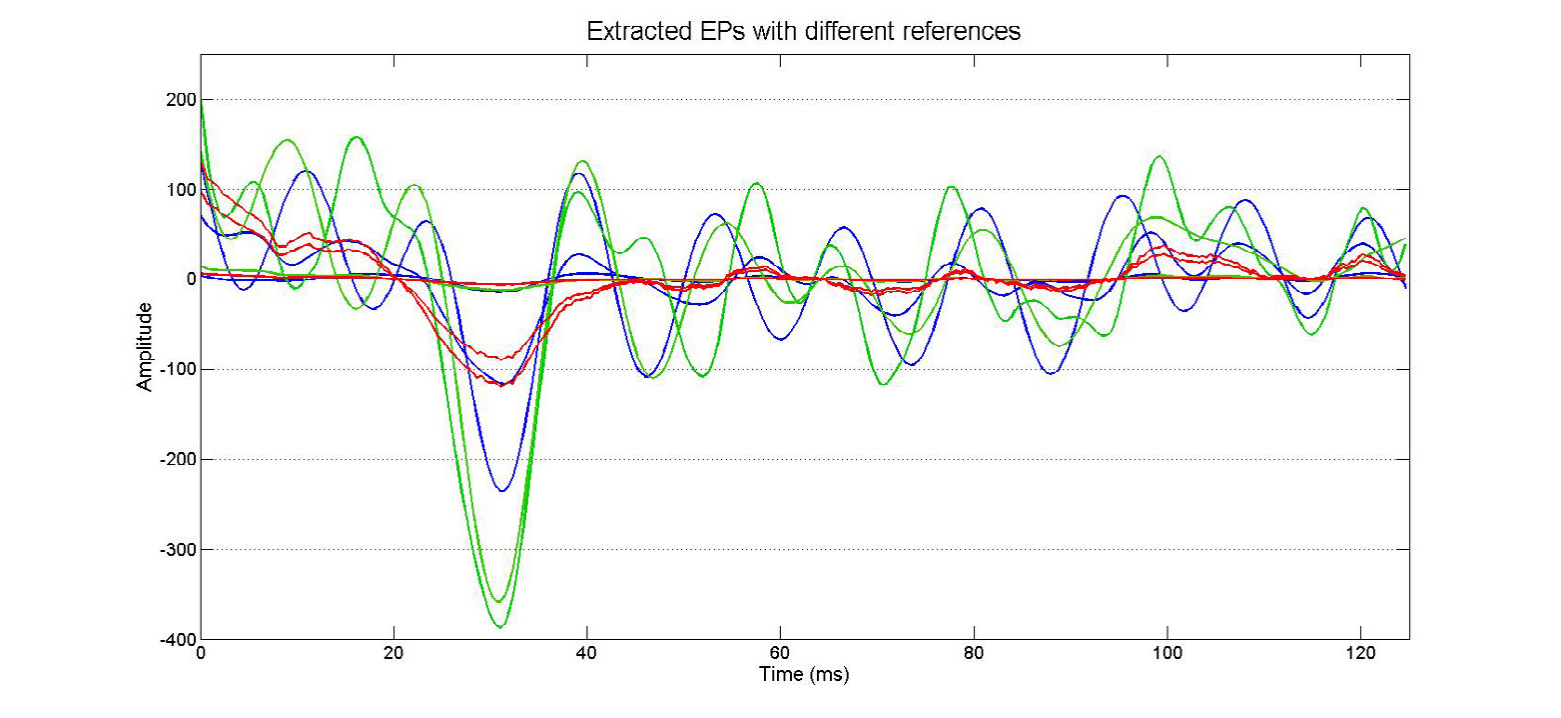
**Simulation results**. The target input signals for the 50^th^, 51^st^, 52^nd ^trial with their 3 different amplitudes (100%, 80%, 5%) are plotted in red. The EP estimated using the ARX with a reference obtained averaging the last 50 trial are plotted in green. The EP obtained using a moving average with a forgetting coefficient is plotted in blue. The amplitude is a generic value.

The 52^nd ^EP input is correctly extracted from the raw signal by the ARX filter both using, as input model reference, the average of the previous 50 trials or using the moving average with a forgetting coefficient *μ *= 0.95 (Fig. 4).

However the difference due to the reference is clearly visible in the extraction of the 51^st ^trial. The 51^st ^raw trial, as described above, is built from an EP reduced by 20% in respect to the EP of the 50^th ^trial. Utilizing the moving average with a forgetting coefficient, it is possible to notice a variation of extracted EP of about 50% on P30 peak amplitude whereas using as reference the average of the previous 50 trails the peak shows a decrease of only 5%.

**Figure 5:**
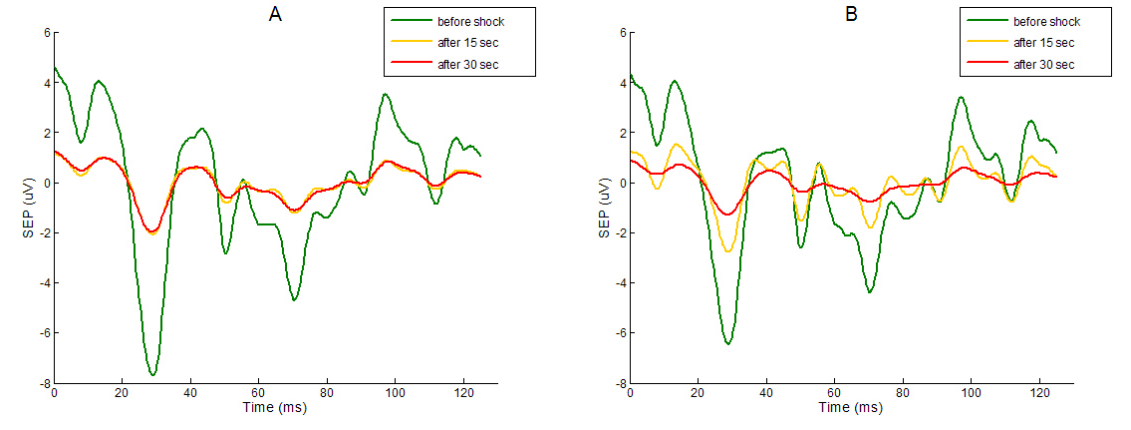
**EPs after a sequence of hammer blows obtained by the ARX filtering with two different reference signals**. In A the EPs are obtained using as the reference signal the moving average with forgetting coefficient. In B the EPs are obtained using the average of the last 50.

Three significant trials have been chosen from one of the eight cases studied (described in further detail in the next section) in order to show how the reference signal, chosen for the ARX filter, affects the tracking of the changes in the SEP waveform after a sequence of hammer blows at the vertebral column (Fig. 5). Using the exponential average with the forgetting factor as ARX reference signal (Fig. 5A) the tracking of the sudden waveform change is faster than using the classical moving average (Fig. 5B). This increased sensibility to the EP variation is also observed in the signal processing simulation previously presented (Fig. 4).

Considering the correlation of the P30 EP amplitude variation with respect to that of the H-reflex one, the Pearson's coefficient shows that in all of the 8 cases analyzed the two sets of data are correlated with a confidence level p < 0.01 (Table 2).

**Table 2:** Degree of correlation between SEP and H-reflex obtained computing the Pearson's coefficient.

age	sex	level	**r**	df	p = 0.05	P = 0.01
31	F	D5-L4	**0.501**	31	0.351	0.447
13	F	D5-L1	**0.551**	30	0.355	0.456
17	M	D3-L3	**0.746**	36	0.321	0.413
20	F	D5-L3	**0.611**	47	0.282	0.365
14	F	D4-L4	**0.580**	30	0.355	0.456
17	F	D3-L1	**0.617**	40	0.304	0.393
16	F	D3-L4	**0.489**	41	0.299	0.386
53	F	D4-L5	**0.385**	42	0.294	0.379

The Pearson's coefficient is r, the freedom degree is df, p is the confidence level of test. When the Pearson's coefficient r is greater than the value reported to the column p, there is a correlation with a confidence level of p = 0.05 or p = 0.01. The correlation between the SEP and H-reflex is verified both for p = 0.05 and p = 0.01 for all the cases studied.

### A surgery case

The procedure followed in the signal processing method is here outlined using data obtained from one of the eight cases presented in Table 1.

**Figure 6:**
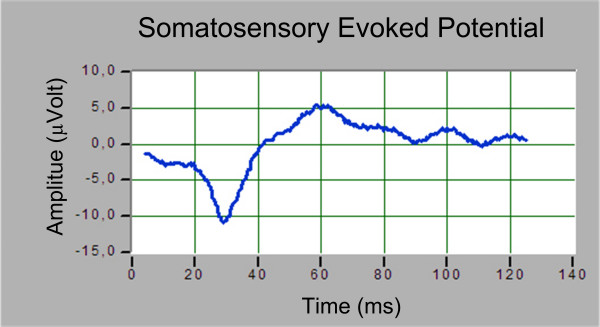
Reference signal obtained at the start of surgery session monitored.

**Figure 7:**
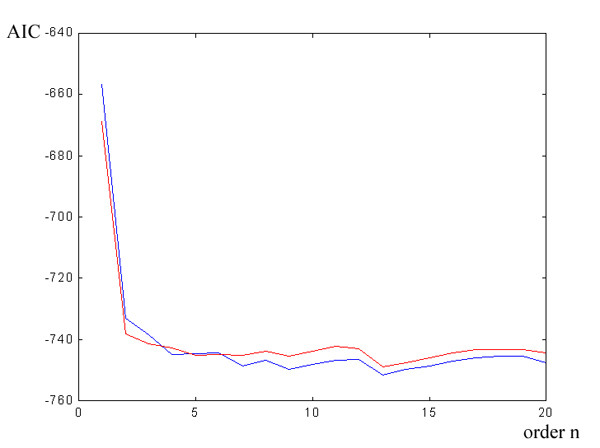
**AIC trend vs. the model order n**. The red plot is with m = 2 and the blue one is with m = 4.

The initial ARX reference signal has been built using 50 trials recorded before the beginning of the surgical procedures (Fig. 6). Reference values, specific to the subject, are obtained from the evaluation of the SEP main peak amplitude (P30) and its latency. The optimum model order is then chosen analyzing the AIC function: Fig. 7 shows the trend of AIC when n varies between 1 and 10 and for two different values of m, m = 2 and m = 4. The optimum values in this case are: n = 2, m = 4 and d = 2.

**Figure 8:**
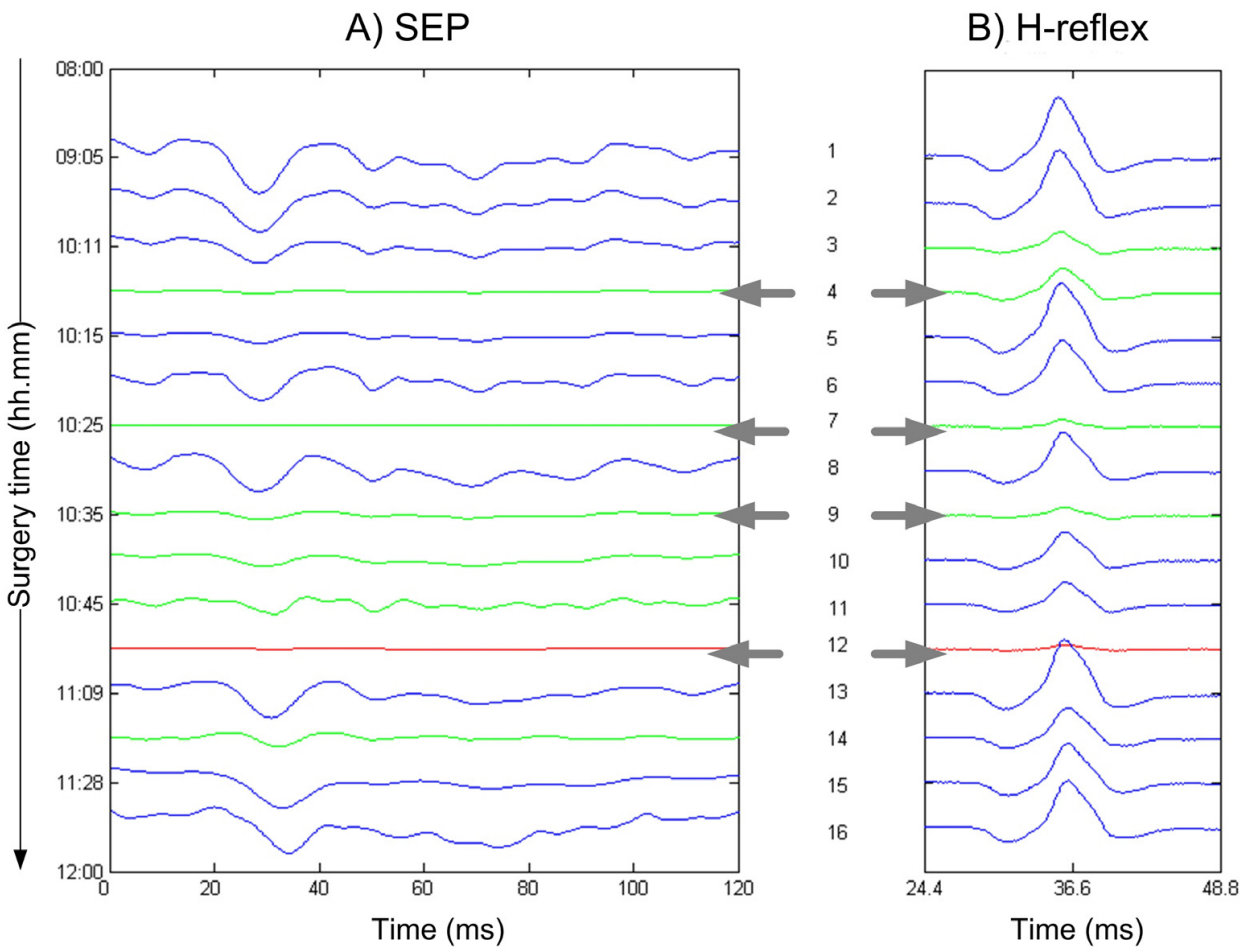
**Surgery session analysis combining the SEP with H-reflex**. The time is in hour:minutes AM. The detailed surgical steps are here presented: 1 09:05 Initial EP an H-Reflex 2 10:00 The EP and H-Reflex are still unmodified 3 10:11 Risky surgery phase is started **4 10:13 After hammer blows: EP and H-reflex have reduced amplitudes **5 10:15 H-reflex is increased but EP amplitude is still low 6 10:21 EP and H-reflex are normal **7 10:25 After hammer blows: EP and H-reflex are weak **8 10:30 EP and H-reflex are normal **9 10:35 Vertebral column traction: EP and H-reflex have very low amplitudes **10 10:39 After traction: very slow amplitude increasing especially for EP 11 10:45 New traction: EP amplitude is very low **12 10:54 After traction: EP is absent and the H-reflex is low **13 11:09 EP and H-reflex are normal 14 11:24 After hammer blows: EP and H-reflex have low amplitudes 15 11:28 Risky surgery phase ended, EP and H-flex are normal 16 11:50 Surgery end, EP and H-flex are normal

Fig. 8a shows a sequence of single trial SEPs during different surgery phases, while in Fig. 8b the corresponding H-reflex responses are plotted. The surgery history summary can be observed in Table 3.

**Table 3:** Surgery history

**Time**	**Maneuvers**
8:44	Incision
10:31	Hammered
10:50	Hammered
11:13	Derotation
11:18	Decortications
11:58	Suture
12:12	End

The time is in hour.minutes AM.

In step 1 and step 2 there is no detectable changes in the monitored waveforms as they are recorded during the initial surgery phase and no stressful maneuvers have been performed yet.

At step 3 the vertebral column begins to be hammered and both signal amplitudes decrease considerably. The two signals, however, show different behaviors. The H-reflex recovers very quickly, whereas the SEP shows a slower recovery dynamic. The same behavior can be observed after other stressful maneuvers, as decortications and tractions.

**Figure 9:**
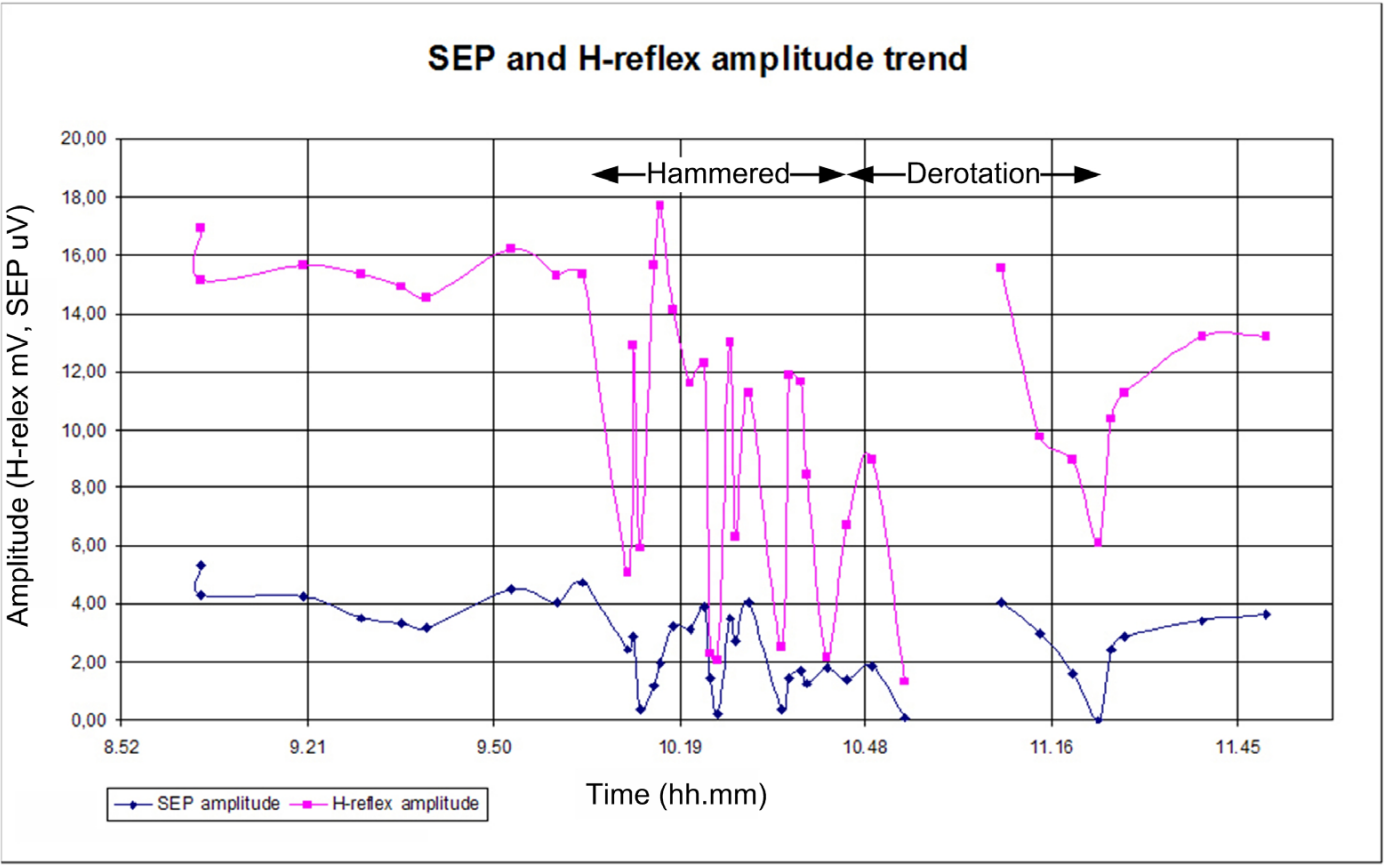
**SEP and H-reflex amplitude trend**. The red plot is the H-reflex and the blue one is the SEP. The amplitude is normalized, the SEP value is divided by 10^-6 ^and the H-reflex is divided by 10^-3^. The time is in hour.minutes AM.

Fig. 9 shows in better detail the amplitude trends of the two signals. As observed in Fig. 8 and 9, both signals are affected by surgical maneuvers such as hammer strokes and decortications. However, the amplitude of the H-reflex restores quickly to the initial values, while the SEP amplitude requires a longer recovery time and does not restore completely, showing a globally decreasing trend.

## Conclusions and discussion

The application target was to implement a signal processing method able to monitor single trial SEPs during surgery and to be very sensitive to the evoked potential variations. The ARX filter, using a reference generated by a recursive exponential averaging, fulfills the requirements and provides better performances than the classical moving average reference. In order to test the method we used a lower high pass filter (1 Hz) than the one utilized in usual neuromonitoring applications (30 Hz) in order to test the signal processing method in less favorable conditions and demonstrating its capability to detect also small single potential variations.

The paper presents off-line results, but the method is designed for online application. During surgery, the time available to complete a single trial extraction is 10 sec, i.e the interstimuli interval. The proposed algorithm completes the analysis in less than 1 sec (running on Laptop with Intel P4 512MB) so it is suitable for online application. Another critical point is the choice of the model orders (n, m, d). The inter-subject variability imposes the calculation of the optimal orders for each new patient as described in the method section and this procedure has to be completed between the beginning of the surgery, when the patient falls asleep, and the starting of the surgical risky maneuvers (about 15 minutes). Actually this procedure is completed in less than 2 minute because, as described in the method section, it needs only 50 trials obtained at higher stimulation frequency (5 Hz). There is also some intra-individual variability, even if less critical than inter-subject variability. For this reason the test of the model is carried out each trial through the whiteness test of the prediction error err(k). In the event of a not whiteness of the err(k) the model orders are increased.

Other signal processing methods [29,30] have been proposed in literature for SEP monitoring during surgery, but the presented application compared the single trial SEP with the H-reflex using the same stimulus delivered at very low frequency. For this reason we develop a signal processing method, based on previous works [22-26], which gives information at each single trial recorded. The method improves the sensibility to the SEP variation as presented in the result section. Though 8 surgery sessions are not enough to have clinical and medical results, some comments are still possible, even at this early stage of research. The analysis of these 8 clinical cases recorded engenders a general consideration about the correlation between SEP and H-reflex amplitudes that are statistically correlated as shown by the Pearson's coefficient. As expected, the Pearson's coefficient shows a fair correlation between the amplitude of the single trial SEP and the H-reflex. As described previously, the behavior of the two signals is quite different regarding the response of the spinal cord to a stressful maneuver. However, a correlation exists between the EP and H-reflex. The example above, in fact, shows that the same maneuver affects both signals. The presence of correlation between the changes that occur in both signals means that the changes are related to the same cause: in this case, this could mean that modification of the spinal cord functionality is induced by the same surgical maneuvers. Thus, the two signals, anatomically and functionally independent, generated by a single transcutaneous stimulus, are both related to the functionality of the spinal cord and are both affected by the same cause. Regarding the neuromonitoring technique where the method has been applied, the combined neuromonitoring has shown the expected behavior: even if SEPs and H-reflex monitor anatomically different pathways, when they are affected by the same surgical maneuvers, they show significantly correlated amplitude changes.

A larger set of monitored surgeries will be collected in order to obtain a strong and significant clinical result that confirms a higher reliability of this new combined monitoring technique.

## Authors' contributions

FB proposed the neuromonitoring technique and was in charge of the neuromonitoring in the operating theater. SC proposed the signal processing method. AMB and AM performed the signal processing and statistical analysis. LR worked on the simulation of the off-line data processing. AG helped to record data in surgery sessions.

All authors read and approved the final manuscript.
